# Supplementary material for: The Ratio of GrzB+ − FoxP3+ over CD3+ T Cells as a Potential Predictor of Response to Nivolumab in Patients with Metastatic Melanoma
Source: Cancers (Basel). 2021 May 12;13(10):2325. doi: 10.3390/cancers13102325 (PMC8150779; doi:10.3390/cancers13102325)
Supplement: Supplementary file 1 [file cancers-13-02325-s001.zip › cancers-1153411-supplementary.pdf]

Supplementary Table S1. Antibody specifications. Primary and secondary antibodies; manufacturers and incubation conditions for primary antibodies; detection systems.

Marker	Host	Clone	Epitope Retrieval	Dilution	Company	Procedure	automated immunostainer	Detection systems/ Secondary antibodies
CD3	Rabbit	2GV6	CC1	prediluted	Ventana, Roche	automate	BenchMark XT autostainer (Ventana, Roche)	Ventana ultraView Universal DAB Detection Kit (ref. 750-600)
CD8	Mouse	C8/144b	CC1	1/100	Dako	automate	BenchMark XT autostainer (Ventana, Roche)	Ventana ultraView Universal DAB Detection Kit (ref. 750-600)
FOXP3	Rabbit	D2E8E	Ph 8	1/150	Cell Signaling Technology	manual	not	Goat anti-rabbit HRP conjugated (SK001 Dako)
GRZB	Rabbit	polyclonal	Ph 8	1/100	Abcam (ab4059)	manual	not	Goat anti-rabbit HRP conjugated (SK001 Dako)
PD-L1	Rabbit	E1L3N	PH8	1/200	Cell Signaling Technology	manual	not	Goat anti-rabbit HRP conjugated (SK001 Dako)
MMR-MLH1	Mouse	ES05	PH6	1/100	Leica/Novocastra	automate	Bond III (Leica Biosystems)	Leica Bond Polymer Refine Detection (ref. DS9800)
MMR-MSH2	Mouse	25D12	PH6	1/200	Leica/Novocastra	automate	Bond III (Leica Biosystems)	Leica Bond Polymer Refine Detection (ref. DS9800)
MMR-MSH6	Mouse	PU29	PH6	1/200	Leica/Novocastra	automate	Bond III (Leica Biosystems)	Leica Bond Polymer Refine Detection (ref. DS9800)
MMR-PMS2	Mouse	MORG4	PH8	1/100	Leica/Novocastra	automate	Bond III (Leica Biosystems)	Leica Bond Polymer Refine Detection (ref. DS9800)
